# Assessment of tumor treatment response using active contrast encoding (ACE)-MRI: Comparison with conventional DCE-MRI

**DOI:** 10.1371/journal.pone.0234520

**Published:** 2020-06-10

**Authors:** Jin Zhang, Kerryanne Winters, Karl Kiser, Mehran Baboli, Sungheon Gene Kim

**Affiliations:** Department of Radiology, Center for Biomedical Imaging, New York University School of Medicine, New York, New York, United States of America; Henry Ford Health System, UNITED STATES

## Abstract

**Purpose:**

To investigate the validity of contrast kinetic parameter estimates from Active Contrast Encoding (ACE)-MRI against those from conventional Dynamic Contrast-Enhanced (DCE)-MRI for evaluation of tumor treatment response in mouse tumor models.

**Methods:**

The ACE-MRI method that incorporates measurement of *T*_*1*_ and *B*_*1*_ into the enhancement curve washout region, was implemented on a 7T MRI scanner to measure tracer kinetic model parameters of 4T1 and GL261 tumors with treatment using bevacizumab and 5FU. A portion of the same ACE-MRI data was used for conventional DCE-MRI data analysis with a separately measured pre-contrast *T*_*1*_ map. Tracer kinetic model parameters, such as *K*^*trans*^ (permeability area surface product) and *v*_*e*_ (extracellular space volume fraction), estimated from ACE-MRI were compared with those from DCE-MRI, in terms of correlation and Bland-Altman analyses.

**Results:**

A three-fold increase of the median *K*^*trans*^ by treatment was observed in the flank 4T1 tumors by both ACE-MRI and DCE-MRI. In contrast, the brain tumors did not show a significant change by the treatment in either ACE-MRI or DCE-MRI. *K*^*trans*^ and *v*_*e*_ values of the tumors from ACE-MRI were strongly correlated with those from DCE-MRI methods with correlation coefficients of 0.92 and 0.78, respectively, for the median values of 17 tumors. The Bland-Altman plot analysis showed a mean difference of -0.01 min^-1^ for *K*^*trans*^ with the 95% limits of agreement of -0.12 min^-1^ to 0.09 min^-1^, and -0.05 with -0.37 to 0.26 for *v*_*e*_.

**Conclusion:**

The tracer kinetic model parameters estimated from ACE-MRI and their changes by treatment closely matched those of DCE-MRI, which suggests that ACE-MRI can be used in place of conventional DCE-MRI for tumor progression monitoring and treatment response evaluation with a reduced scan time.

## Introduction

*T*_*1*_-weighted dynamic contrast-enhanced magnetic resonance imaging (DCE-MRI) is one of the quantitative MRI methods that have been widely used to assess tumor treatment response [1–5]. DCE-MRI with tracer kinetic model analysis provides physiologically relevant parameters, such as transfer constant (*K*^*trans*^), plasma volume fraction (*v*_*p*_), and extravascular extracellular space volume fraction (*v*_*e*_) [6, 7]. These parameters are closely linked to tumor biology. They can be used as surrogate markers of anti-angiogenic and cytotoxic responses that are more sensitive to subtle internal tumor responses than volume changes [8]. Recent development of various fast MRI acquisition methods has helped advance the field of DCE-MRI with opportunities to correct for motion artifacts [9, 10] and to acquire a high temporal resolution without compromising the spatial resolution [11, 12]. However, extracting quantitative measures from DCE-MRI data remains nontrivial in practice, partly due to the uncertainties in some of the input data required for the tracer kinetic model analysis, such as arterial input function [13], relaxivity of Gd-based contrast agent in tissue [14], longitudinal relaxation rate constant (*T*_*1*_) of the tissue [15], and the transmit RF field inhomogeneity (*B*_*1*_) [16].

*T*_*1*_ value before contrast injection, often referred to as baseline *T*_*1*_ (*T*_*10*_), is one of the essential parameters for quantitative tracer kinetic model analysis. *T*_*10*_ of a target tissue is used in the conversion of a time-intensity curve from *T*_*1*_-weighted DCE-MRI data to the time-concentration curve of contrast agent in the tissue. It has been shown that the accuracy of *T*_*10*_ measurement is directly related to the accuracy of tracer kinetic model parameters; underestimation of *T*_*10*_ values can result in overestimation of *K*^*trans*^ and *v*_*e*_, and overestimation of *T*_*10*_ in underestimation of those kinetic parameters [17]. Hence, it is crucial to have accurate *T*_*10*_ measures, particularly for longitudinal studies as well as multi-site studies, to minimize the influence of errors in *T*_*10*_ on the measurement of the kinetic parameters of interest [18].

One of the most commonly used methods for *T*_*1*_ mapping is the variable flip angle (VFA) method with three to five different flip angles [18]. It has been shown that the VFA protocols with such small number of flip angles could be not as robust as the inversion-recovery methods in terms of test-retest repeatability, but still could be optimized to have an acceptable balance of accuracy and repeatability with errors less than 15% [15]. One of the major advantages of using the VFA method for *T*_*1*_ mapping is that the scan time can be substantially shorter than that required for an inversion-recovery based approach. However, if the image matrix is large, for instance in breast MRI, the scan time to acquire images for each flip angle can still be considerably long (typically > 1 min), making the total time required for *T*_*1*_ mapping a significant portion of the scan time. Extra time and effort needed for *B*_*1*_ correction in *T*_*1*_ mapping is also an additional challenge in quantitative DCE-MRI studies.

To address this challenge of having to spend additional scan time to measure *T*_*10*_ before the actual DCE-MRI scan, a novel imaging framework, referred to as Active Contrast Encoding (ACE)-MRI, has been proposed. ACE-MRI integrates *T*_*1*_ mapping into the wash-out part of the dynamic scan after contrast injection such that *T*_*10*_ can be estimated simultaneously with the tracer kinetic model parameters from the same contrast enhancement data [19]. It was successfully demonstrated that the tracer kinetic model parameters estimated from the ACE-MRI method matched well with those from a conventional DCE-MRI approach with separate measurement of *T*_*10*_ maps. However, it was not shown whether the ACE-MRI method can be used to evaluate tumor treatment response and also provide the same changes of the tracer kinetic model parameters as measured in the conventional DCE-MRI method with separate measurement of *T*_*10*_ map. Hence, the purpose of this study was to evaluate tumor treatment response using ACE-MRI, and to compare the ACE-MRI results with those from the conventional DCE-MRI method, using mouse models of brain tumor and breast cancer.

## Methods

### Animal models

This study used two mouse tumor models for comparison of treatment responses measured in terms of the tracer kinetic model parameters from ACE-MRI and DCE-MRI. For a breast cancer model, six to eight-week-old BALB/c mice (Taconic Biosciences, Albany, NY; *n* = 13, labeled as M1-M13 in this study) were given a subcutaneous injection of 1 × 10^5^ 4T1 murine breast cancer cells (ATCC, Manassas, VA) suspended in 0.1mL of phosphate-buffered saline (PBS) in the right flank (9 mice, M1-M9) or a stereotaxic intracranial injection of 1 × 10^6^ cells suspended in 0.004mL of PBS using a Hamilton syringe (4 mice, M10-M13). In addition, six to eight-week-old C57BL6 mice (Taconic Biosciences, Albany, NY; *n* = 4, M14-M17) were given a stereotaxic intracranial injection of 1 × 10^6^ GL261 mouse glioma cells (ATCC, Manassas, VA) suspended in 0.004mL of PBS. Each mouse was scanned twice, starting post-injection days 10–38 when the tumor size was 2 mm or larger in diameter; the first scan for pre-treatment and the second scan after treatment. The two mouse tumor models described above, were used to compare ACE-MRI and DCE-MRI in the assessment of treatment response.

For the treatment group (*n* = 8 with four 4T1 flank tumors, three 4T1 brain tumors and a GL261 brain tumor), pre-treatment MRI was followed by an intraperitoneal (IP) injection of bevacizumab (10 mg/kg) on the same day and an IP injection of 5FU (80mg/kg) the following day. Post-treatment MRI was conducted about 24 hours after the 5FU treatment (i.e., two days after the pre-treatment MRI). The control group (*n* = 9 with five 4T1 flank tumors, one 4T1 brain tumor, and three GL261 brain tumors) was treated with sodium chloride solution (10 ml/kg) and imaged at the same time points as the treatment group. All mice were housed in cages with filter cage tops. When the cage tops needed to be opened, it was performed under a hood, one cage at a time. Food and water were available *ad libitum*, and the housing room was maintained on a 12-h light-dark cycle (lights on at 07.00h). Following the final scan, the mice were euthanized using exposure to carbon dioxide and cervical dislocation. The mice were treated in strict accordance with the National Institutes of Health Guide for the Care and Use of Laboratory Animals, and this study was approved by the Institutional Animal Care and Use Committee at New York University School of Medicine.

### Data acquisition

MRI experiments were performed on a 7T micro-MRI system, consisting of a Biospec Avance II console (Bruker Biospin MRI, Ettlingen, Germany) interfaced to a 200-mm horizontal bore magnet (Magnex Scientific, Yarnton, Kidlington, Oxfordshire, UK) with an actively shielded gradient coil (Bruker, BGA-95; gradient strength, 750 mT/m). A quadrature Litz body coil (inner diameter 32mm) (Doty Scientific, Columbia, SC, USA) was used to image the 4T1 tumors on the flank, and an in-house built quadrature Litz brain coil (inner diameter 21mm) was used to image the animals with intracranial brain tumors. General anesthesia was induced by 1.5% isoflurane in air. The animals were mounted on a cradle with respiratory and temperature monitoring probes. The animal body temperature was maintained at 34 ± 2 ºC during the scan.

For ACE-MRI, both flip angle (*FA*) and repetition time (*TR*) were varied during the dynamic scan to have active contrast encoding [19], such that the dynamic scan includes multiple segments with different *T*_*1*_/*B*_*1*_-weighted contrasts. In this study, ACE-MRI protocol was implemented using a 3D FLASH sequence with 9 segments; *FA* = {10°, 20°, 5°, 10°, 30°, 2°, 10°, 80°, 10°} and *TR* = {12, 12, 12, 12, 12, 12, 12, 100, 12} ms for {40, 5, 5, 5, 5, 5, 5, 3, 5} frames, respectively. *TE* was kept at 3.83 ms for all segments. Temporal resolution was 5.4 s/frame for small flip angles (*FA* < 80^o^) and 45 s/frame for *FA* = 80^o^. This encoding scheme was selected to have multiple small flip angles around the Ernst angle with the given *TR* and an approximate range of *T*_*1*_ values expected in this study at 7T (1.5–2.5 s). This scan protocol was run to acquire 78 3D images for about 10 min in total.

The baseline protocol (*FA* = 10° and *TR* = 12 ms) was repeated multiple times intermittently to allow interpolation of the time intensity curve between the data with the baseline protocol so that the data with the baseline protocol can be used for the conventional DCE-MRI with a fast sampling during the wash-in phase and intermittent samplings during the wash-out phase. This scheme allows us to use one dynamic scan data with a single injection for both analyses; ACE-MRI with the entire data set (all nine segments) and DCE-MRI using only the data with the baseline protocol (four segments with *FA* = 10^o^ and *TR* = 12 ms).

For the flank tumors, the scan protocol was set to have data acquisition matrix = 100 x 50 x 9 and image matrix = 100 x 66 x 9 with zero padding in the phase encoding direction, FOV = 30 x 20 x 9 mm^3^, and spatial resolution = 0.3 x 0.3 x 1 mm^3^. For the brain tumors, data acquisition matrix = 100 x 50 x 9 and image matrix = 100 x 100 x 9 with zero padding in the phase encoding direction, FOV = 20 x 20 x 9 mm^3^, and spatial resolution = 0.2 x 0.2 x 1 mm^3^. A bolus of Gd-DTPA in saline at the standard dose of 0.1 mmol/kg was injected through a tail vein catheter, starting 1 min after the start of the dynamic scan. For the conventional DCE-MRI data analysis, *T*_*10*_ was separately measured using the RARE-VTR (Rapid Acquisition with Relaxation Enhancement—Variable TR) pulse sequence provided by the scanner manufacturer. *B*_*1*_ mapping was not included in this study based on our previous study that showed less than 10% change across the mouse brain and *B*_*1*_ was assumed as 1 for the DCE-MRI data analysis. *T*_*1*_ mapping using the RARE-VTR took about 11 min. Hence the total scan time for the conventional DCE-MRI study with pre-contrast *T*_*1*_ mapping was 20 min.

### Data analysis

The tracer kinetic model parameters were estimated from the measured data *S*_*m*_*(t)* using the following method. Arterial input function (AIF) was generated with a reference tissue approach [20] using a muscle (spinal muscle for flank tumors and masseter muscle for brain tumors) as a reference tissue with assumed parameters *K*^*trans*^ = 0.11 min^-1^ and *v*_*e*_ = 0.20. AIF estimation was conducted with the baseline protocol data. The relaxivity of the contrast agent, *r*_*1*_, was assumed as 4.3 mM^-1^S^-1^. The tissue concentration, *C*_*t*_*(t)*, was estimated with the modified generalized kinetic model (GKM) [6]:

$$C_{t}(t)=v_{p}C_{p(t)}+K^{trans}\int_{0}^{t}C_{p}(u)e^{\left(-\frac{K^{trans}}{v_{e}}(t-u)\right)}du$$ [1]

The tissue concentration was converted to the longitudinal relaxation rate under the assumption of the fast exchange limit, *R*_*1*_*(t) = 1/T*_*1*_ *+ r*_*1*_*C*_*t*_*(t)*, with *R*_*1*_*(t)* is contrast agent relaxivity. Then, the predicted signal intensity *S*_*p*_*(t)* was calculated using the spoiled gradient-echo sequence with *R*_*1*_*(t)*, *FA(t)* and *TR(t)*:

$$S_{p}(t)=S_{0}\frac{(1-e^{-R_{1}(t)TR(t)})\sin\left(B_{1}FA(t)\right)}{\left(1-{\cos\left(B_{1}FA(t)\right)e}^{-R_{1}(t)TR(t)}\right)}e^{-\frac{TE}{T_{2}^{*}}}$$ [2]

where *S*_*0*_ represents the fully relaxed signal for a 90° pulse when *TR* >> 1/*R*_*1*_*(t)* and *TE* << *T*_2_^*^.

For conventional DCE-MRI analysis, the data collected for the time points with the baseline protocol (*T*_*bp*_) were used for tracer kinetic model analysis, together with independently measured pre-contrast *T*_*10*_. *B*_*1*_ assumed to be 1 for all voxels. Hence, the tracer kinetic model parameters were estimated from the measured signal intensity *S*_*m*_*(t)* as the following:

$$\{K^{trans},v_{e},v_{p}\}=\arg\;\min\sum_{t\in T_{bp}}{\left(\frac{S_{m}(t)}{S_{m}(0)}-\frac{S_{p}(t)}{S_{p}(0)}|B_{1},T_{10}\right)}^{2}$$ [3]

In contrast, the data analysis with the ACE-MRI approach was conducted with all data points with multiple *FA* and *TR* values to estimate *T*_*10*_ and *B*_*1*_ along with the tracer kinetic model parameters simultaneously [19]:

$$\{K^{trans},v_{e},v_{p},B_{1},T_{10}\}=\arg\;min\sum_{\forall t}{\left(\frac{S_{m}(t)}{S_{m}(0)}-\frac{S_{p}(t)}{S_{p}(0)}\right)}^{2}.$$ [4]

The parameter estimation was conducted using the Simplex [21] method provided in Matlab (MathWorks, Natick, MA) with parameters were limited within physiologically relevant range (*K*^*trans*^: 0.001–2.0 min^-1^, *v*_*e*_: 0.001–1.0, *v*_*p*_; 0.001–0.5 and *T*_*1*_: 0.001–4.0 s) by using constrained optimization. For each data set, the model fit was repeated with 20 randomly selected initial guesses to find the best fit with the least sum of squared differences.

### Statistical analysis

Initial Area Under the Curve (IAUC) [22] was used to select the enhancing tumor voxels for comparison between the ACE-MRI and DCE-MRI methods. Bland-Altman plots [23] and Pearson correlation were used to evaluate the agreement between the tracer kinetic model parameters measured from the ACE-MRI and DCE-MRI methods. Treatment response was assessed by the changes of pharmacokinetic model parameters estimated before and after treatment. These parameters were compared using the Wilcoxon rank sum test. All statistical tests were conducted at the two-sided 5% significance level.

## Results

**Fig 1:**
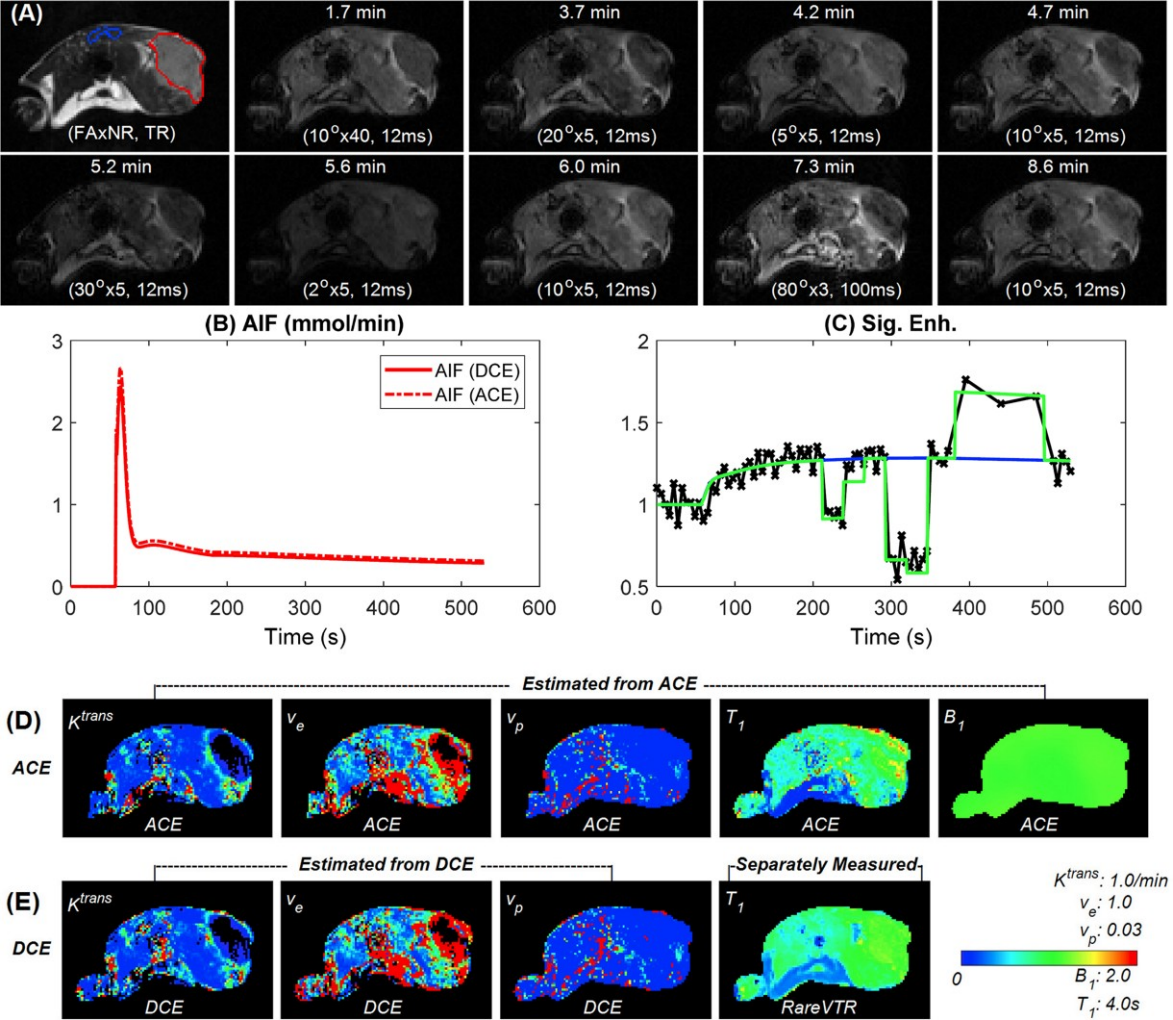
A representative example (4T1 flank tumor) of pharmacokinetic model parameter estimation using ACE-MRI and DCE-MRI protocol. (a) Demonstration axial images at different ACE-MRI encoding stages with reference muscle ROI (blue) and tumor ROI (red) overlay on *T*_*2*_ weighted image. (b) AIF functions from reference tissue method for ACE-MRI (dashed line) and DCE-MRI (solid line). (c) Demonstration tumor voxel enhancement curve (black cross) with ACE-MRI fitting (green solid) and DCE-MRI fitting (blue solid). (d) *K*^*trans*^, *v*_*e*_, *v*_*p*_, *T*_*1*_ and *B*_*1*_ parameter maps simultaneously estimated with ACE-MRI protocol. (e) *K*^*trans*^, *v*_*e*_ and *v*_*p*_ parameter maps estimated with DCE-MRI protocol with separately measured *T*_*1*_ map using the RARE-VTR method.

Fig 1 shows a representative example of a 4T1 tumor for tracer kinetic model analysis with the ACE-MRI and DCE-MRI methods. The ACE-MRI method produces images with different contrasts for the segments with varying combinations of *FA* and *TR*, i.e. active encoding (Fig 1A). The change of the signal intensity with the active encoding can also be observed clearly in the signal enhancement curve of one tumor voxel shown in Fig 1C. The tracer kinetic model fit can be done using only the data acquired with the baseline protocol, the separately measured *T*_*10*_ value and assumed *B*_*1*_ = 1 for the conventional DCE-MRI data analysis, which produces a continuous enhancement curve (blue line in Fig 1C) that is typically observed in DCE-MRI studies. For ACE-MRI, the model fit to the signal enhancement curve would have jumps to account for the active contrast encoding with different *FA* and *TR* combinations (green line in Fig 1C). The AIF curves of both ACE-MRI and DCE-MRI were estimated from a reference region in the muscle (Fig 1B). Note that the AIF concentration curve of ACE-MRI is continues without jumps; this is also the case for any tissue voxel where the jumps are only observed in the signal curves with the active contrast encoding. The tracer kinetic model parameter maps from ACE-MRI match well those from DCE-MRI with separately measured *T*_*10*_ maps, as shown in Fig 1D and Fig 1E.

**Fig 2:**
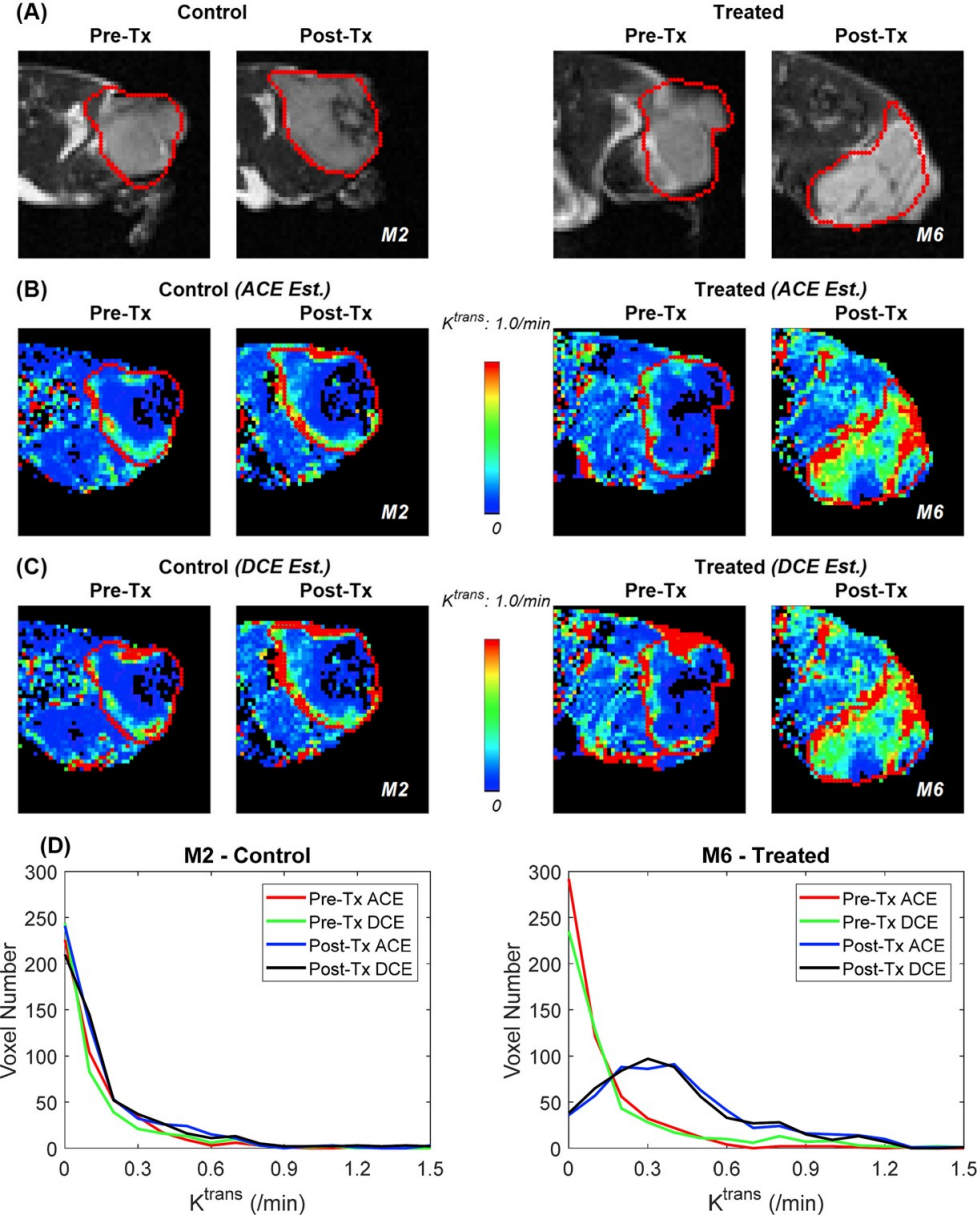
Representative *T*_*2*_ weighted tumor images (a) for control group (mouse M2) and treatment group (M6) at pre-treatment (Pre-Tx) and post-treatment (Post-Tx). *K*^*trans*^ maps of the control group (mouse M2) and treatment group (M6) using the ACE-MRI method (b) and conventional DCE-MRI method (c) at Pre-Tx and Post-Tx scans. Red ROI indicates tumor lesions. (d) Histograms of estimated *K*^*trans*^ values of voxels in the tumors shown in (b) and (c) for M2 and M6.

The pre- and post-treatment *K*^*trans*^ maps for an example case of 4T1 tumors from the control group and another 4T1 tumor from the treatment group are shown in Fig 2. For all cases, the *K*^*trans*^ maps of ACE-MRI are in good agreement with those of DCE-MRI, in terms of the parameter maps and histogram distributions. As shown in these examples, the 4T1 tumors typically had poor contrast enhancement, except in the rim. However, the treatment with one cycle of bevacizumab and 5FU substantially improved the contrast enhancement across the whole tumor. Such treatment response was observed equally with the *K*^*trans*^ maps of both the ACE-MRI and DCE-MRI data.

**Fig 3:**
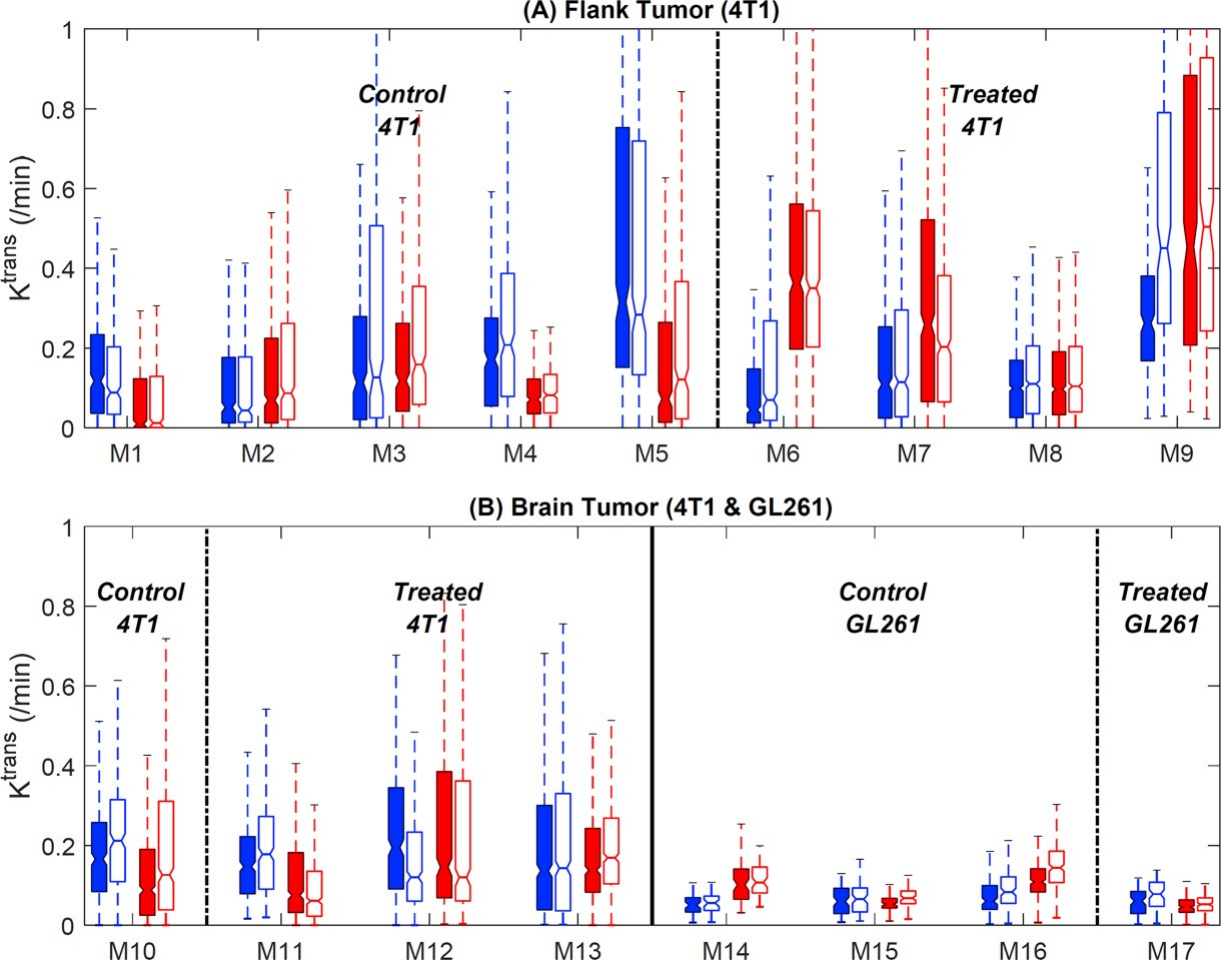
**Comparison of *K***^***trans***^ **values estimated for the voxels in tumor ROIs using the ACE-MRI (filled boxes) and DCE-MRI (open boxes) methods with pre-treatment (blue) and post-treatment (red) data.** (a) Flank tumors (4T1: M1-M9). (b) Brain tumors (4T1: M10-M13 and GL261: M14-M17).

The *K*^*trans*^ values from the tumor voxels of individual animals are summarized using the box plots in Fig 3, which show that there are overall good agreements of the measurement between DCE-MRI and ACE-MRI, without any noticeable bias between the two methods in terms of parameter distribution. The 4T1 tumors both in the flank and brain appear to have a larger range of *K*^*trans*^ values than the GL261 tumors, mainly due to a large difference between the enhancing rim and non-enhancing core regions of 4T1 tumors as shown in Fig 2. In contrast, GL261 tumors had similar contrast enhancement across the lesion with an overall lower level of enhancement, as suggested by smaller *K*^*trans*^ values.

**Fig 4:**
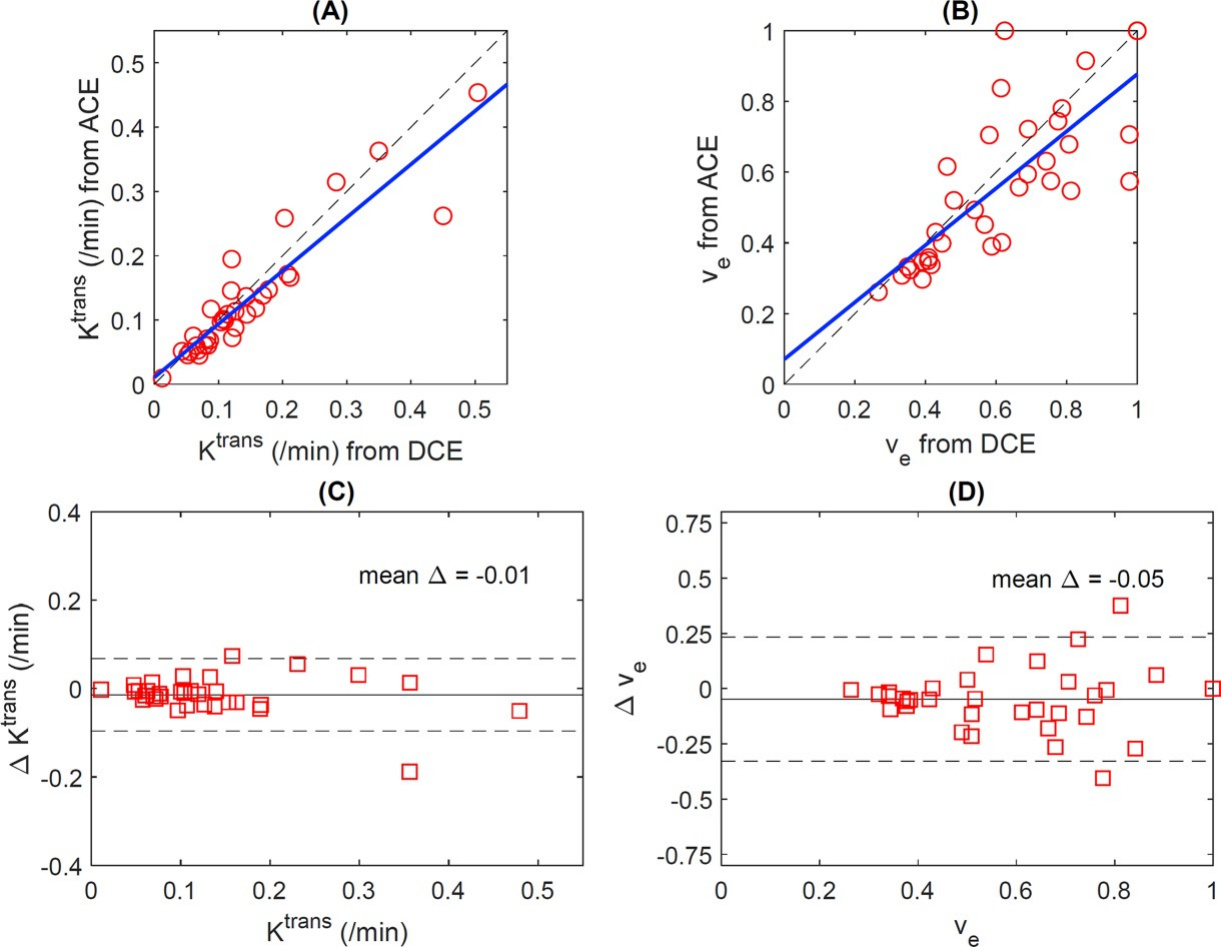
Scatter plots showed the tumor median values comparisons (red circle) between DCE-MRI and ACE-MRI methods for *K*^*trans*^ (a) and *v*_*e*_ (b) for all the tumors of the 17 mice. Blue lines are regression lines and dash lines are lines of unity. The correlation coefficients are 0.92 for (a) and 0.78 for (b) with *p*-values < 0.0001 for both cases. Bland-Altman plots show tumor median value comparisons (red square) between DCE-MRI and ACE-MRI methods for pre and post-treatment for all tumors of the 17 mice: *K*^*trans*^ pre/post-treatment (c) and *v*_*e*_ pre/post-treatment (d). Solid thin lines are the mean difference (actual values are noted in each plot) and dash thin lines are mean Δ ± 1.96 SD.

The scatter plots of the median *K*^*trans*^ values and median *v*_*e*_ values for all the 34 datasets (17 animals with pre and post-treatment for each animal) shows a good agreement between the values of the ACE-MRI and DCE-MRI protocols, with correlation coefficient of 0.92 (*p* < 0.0001) for *K*^*trans*^ (Fig 4A) and 0.78 (*p* < 0.001) for *v*_*e*_ (Fig 4B). Table 1 shows the median values of *K*^*trans*^ and *v*_*e*_ for all the animals before and after treatment using DCE-MRI or ACE-MRI methods. The Bland-Altman plots in Fig 4C for the *K*^*trans*^ and Fig 4D for the *v*_*e*_ show that there is no noticeable trend of data depending on the magnitude of the values, expect one case with a large difference of about -0.2 for *K*^*trans*^. The mean difference of *K*^*trans*^ between DCE-MRI and ACE-MRI remained closed to zero, so did that of *v*_*e*_. The 95% limits of agreement were -0.12 min^-1^ to 0.09 min^-1^ for *K*^*trans*^ and -0.37 to 0.26 for *v*_*e*_. The *v*_*e*_ estimation has a less obvious trend (regression line slope is 0.04) compared to -0.11 for *K*^*trans*^ estimation which was mostly affected by one outlier outside of the limit of agreement as shown in the plot.

**Table 1:** List of median *K*^*trans*^ and *v*_*e*_ values for all the tumors obtained with DCE-MRI and ACE-MRI.

DCE-MRI	DCE-MRI				ACE-MRI			
	*K*^*trans*^ *(min*^*-1*^*)*		*v*_*e*_		*K*^*trans*^ *(min*^*-1*^*)*		*v*_*e*_	
	Pre-Tx	Post-Tx	Pre-Tx	Post-Tx	Pre-Tx	Post-Tx	Pre-Tx	Post-Tx
**M1**	0.088	0.012	1.00	0.81	0.117	0.010	1.00	0.68
**M2**	0.043	0.087	0.48	0.57	0.051	0.069	0.52	0.45
**M3**	0.126	0.158	0.81	0.98	0.113	0.118	0.55	0.71
**M4**	0.208	0.082	0.69	0.41	0.171	0.071	0.59	0.36
**M5**	0.284	0.121	1.00	0.62	0.315	0.072	1.00	0.40
**M6**	0.070	0.350	0.98	0.85	0.045	0.363	0.57	0.92
**M7**	0.114	0.203	0.79	0.61	0.109	0.258	0.78	0.84
**M8**	0.110	0.104	0.39	0.43	0.099	0.097	0.35	0.43
**M9**	0.450	0.504	0.75	0.78	0.262	0.454	0.57	0.74
**M10**	0.056	0.107	0.35	0.36	0.050	0.101	0.33	0.32
**M11**	0.065	0.068	0.33	0.45	0.060	0.053	0.31	0.40
**M12**	0.084	0.144	0.39	0.42	0.060	0.109	0.30	0.34
**M13**	0.212	0.126	0.74	0.59	0.166	0.088	0.63	0.39
**M14**	0.178	0.061	0.66	0.46	0.147	0.075	0.56	0.62
**M15**	0.121	0.120	0.62	0.58	0.195	0.146	1.00	0.70
**M16**	0.078	0.052	0.41	0.27	0.061	0.045	0.35	0.26
**M17**	0.143	0.169	0.69	0.54	0.137	0.138	0.72	0.49

**Fig 5:**
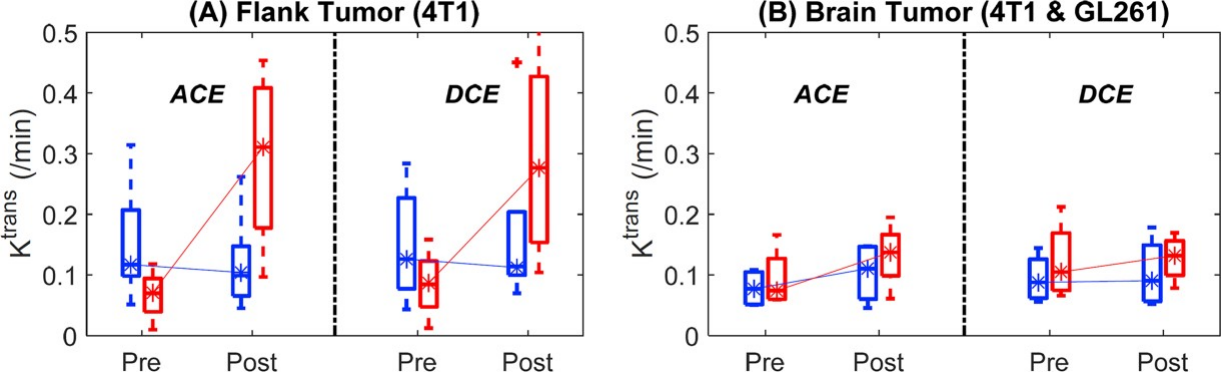
Treatment responses comparison between 4T1 tumors in the flank and 4T1/GL261 tumors in the brain. (a) Median tumor *K*^*trans*^ changes before and after treatment for flank 4T1 tumor. Blue boxplot: control group (*n* = 5), red boxplot: treated group (*n* = 4). (b) Median tumor *K*^*trans*^ changes before and after treatment for brain 4T1/GL261 tumors. Blue boxplot: control group (*n* = 4), red boxplot: treated group (*n* = 4).

Fig 5 shows changes in the tumor median values with and without treatment. In 4T1 flank tumors, the control group did not show any significant change of *K*^*trans*^ between two scans, whereas the treatment group showed about a three-fold increase of *K*^*trans*^ by treatment. In contrast, treatment of the brain tumors induced an increase of *K*^*trans*^ although statistically not significant (*p* = 0.40 for ACE-MRI and 0.70 for DCE-MRI by the Wilcoxon rank sum test). Overall, both DCE-MR and ACE-MRI showed similar trends of *K*^*trans*^ changes by treatment in all tumors.

## Discussion

The results of our study demonstrate that the recently proposed ACE-MRI method can be used to evaluate tumor treatment response without measuring *T*_*10*_ and *B*_*1*_ separately. Tumor growth typically involves the development of structurally and functionally abnormal vascular networks. One of the goals of the anti-angiogenic treatment using bevacizumab is to normalize the vascular structure of the tumors and facilitate the delivery of cytotoxic drugs. In this study, treatment with bevacizumab and 5FU induced increase of *K*^*trans*^, as can be seen from Fig 2B and Fig 2C for one of the 4T1 flank tumor mice and Fig 5 for the whole cohort. In both pre- and post-treatment scans, ACE-MRI and DCE-MRI protocols showed consistent results, which suggests the ACE-MRI can be an efficient tool with a shorter scan time in monitoring the treatment response inside of the tumor.

Treatment with anti-angiogenic drugs combined with conventional cytotoxic drugs [24–29] or immunotherapy [30] is a promising means of treating aggressive cancer. DCE-MRI has been widely used as an important part of most clinical MRI exams for diagnosis of cancer and assessment of treatment response, such that it holds high potential as a single MRI method to estimate both perfusion parameters (such as *K*^*trans*^ and *v*_*p*_) and cellular parameters (such as *v*_*e*_). However, it remains challenging to perform “*quantitative*” DCE-MRI, not to mention additional challenges to reliably measure both perfusion and cellular parameters. One of the practical challenges is to allocate enough scan time for separate measurements of pre-contrast *T*_*1*_ and *B*_*1*_. In addition, the measured *B*_*1*_ and *T*_*10*_ maps often have different fields of view or spatial resolutions such that they are typically co-registered and/or resampled to match the field of view and spatial resolution of the dynamic images for quantitative kinetic model analysis. In ACE-MRI, the *B*_*1*_ and *T*_*10*_ mapping procedures are embedded within the dynamic scan such that there is no extra scan time required to the measurement of *B*_*1*_ and *T*_*10*_ maps and also no need to do additional post-processing to align *B*_*1*_ and *T*_*10*_ maps with the dynamic images. These advantages are demonstrated in this study in the assessment of tumor treatment response.

Assessment of tumor treatment response based on volumetric measures may have severe limitations in terms of assessing therapeutic responses. For example, patients who have measurable disease may show no appreciable change in tumor size even when therapy has successfully halted tumor progression. It is particularly true of new, targeted, non-cytotoxic drugs and when the main purpose of anti-angiogenic treatment is to normalize the tumor vasculature to enhance the delivery of the cytotoxic chemotherapeutic agents. This is demonstrated with the example shown in our study (Fig 2), where the internal change in terms of *K*^*trans*^ in the treated tumor is substantially high, whereas the tumor size appeared about the same within the interval of two days for the pre- and post-treatment scans. It has been reported that bevacizumab can be used to temporarily normalize abnormal vasculature to enable drug delivery and paradoxically increase blood flow and hence drug delivery to tumors [31–33]. Bevacizumab combined with conventional cytotoxic drugs [34], such as 5FU, were used for tumor treatment in this study, with the assumption that bevacizumab can normalize the vasculature [35] which helps 5FU delivery to the tumor. This trend was observed with the 4T1 tumors, but not with the GL261 tumors. However, the same trend was observed with the data analysis results of both DCE-MRI and ACE-MRI.

One of the limitations of the present study is that a separate measurement of *B*_*1*_ map was not conducted. Our previous experience [19] with these small animal imaging coils demonstrated a relatively small variation of the *B*_*1*_ transmit field and the *T*_*1*_ measurement from RARE-VTR is not sensitive to *B*_*1*_ effect. Hence, the DCE-MRI data analysis was conducted with the assumption of *B*_*1*_ = 1 for all voxels, while the ACE-MRI data analysis was conducted with estimated of the *B*_*1*_ field from the data with the active contrast encoding. This could explain some of the differences between DCE-MRI and ACE-MRI observed in this study, such as the differences in individual cases as shown in Figs 3 and 4. This could also be related to the weaker correlation for *v*_*e*_ than for *K*^*trans*^ since *v*_*e*_ is typically more sensitive to *T*_*1*_ and *B*_*1*_ than *K*^*trans*^. However, it was still observed that the tracer kinetic model parameters agreed well between DCE-MRI and ACE-MRI in most cases. Furthermore the overall treatment response did not show a noticeable difference between DCE-MRI and ACE-MRI. *B*_*1*_ mapping for the DCE-MRI analysis, particularly in clinical scanners with a larger field of view, should be included in future studies for more thorough cross-validation of the ACE-MRI approach. A relatively small cohort of animals was another limitation of the study. Future studies should also include the test-retest reproducibility of the ACE-MRI method to establish this as a reliable method to assess tumor treatment response.

In conclusion, the present study demonstrates that the tracer kinetic parameters estimated using ACE-MRI and their changes induced by treatment are in close agreement with those from DCE-MRI and in both 4T1 and GL261 tumors. The results from this study suggest that the ACE-MRI method can be used in place of DCE-MRI, without separate measurement of *T*_*1*_ and *B*_*1*_ maps, for quantitative assessment of tumor progression and treatment response. Future study is warranted with a larger cohort of animals and also to translate the proposed ACE-MRI method for clinical studies.
